# Acute hepatitis of unknown origin in 38-year-old man: A case report

**DOI:** 10.1097/MD.0000000000047041

**Published:** 2026-01-09

**Authors:** Petar Trifonov, Donika Todovichin, Cvetelina Marinova, Anelia Zasheva, Dimitar Komitov, Rosen Nikolov, Raynichka Mihaylova-Garnizova

**Affiliations:** a Clinic of Gastroenterology, UMHAT St. Ivan Rilski, Sofia, Bulgaria; b Clinic of Infectious Diseases, Military Medical Academy, Sofia, Bulgaria.

**Keywords:** acute hepatitis, AHUO, idiopathic hepatitis, unknown

## Abstract

**Rationale:**

Acute hepatitis of unknown origin represents a diagnostic and therapeutic challenge, particularly when common viral, autoimmune, and toxic causes of acute liver injury are excluded.

**Patient concerns:**

We report a case of an adult male. He presents with general fatigue, jaundice, fever, abdominal discomfort. Laboratory findings of severe acute hepatitis and extremely elevated transaminases.

**Diagnoses:**

Full laboratory, serological, and toxicological tests ruled out viral, autoimmune, and drug-induced hepatitis. Metabolic disorders such as Wilson disease and hemochromatosis were also excluded.

**Interventions:**

Supportive therapy consisting of intravenous glucose solutions, silymarin, vitamin C, and ademetionine was initiated.

**Outcomes:**

The patient had rapid clinical improvement, with normalization of liver enzymes at 1-month follow-up.

**Lessons:**

This case shows the importance of a systematic diagnostic approach in acute hepatitis of unknown origin. It suggests that, in adult patients, conservative management with supportive therapy may lead to full recovery despite severe biochemical abnormalities.

## 1. Introduction

Acute hepatitis is an inflammation of the liver, most commonly caused by viral infections (hepatitis A, B, C, D, and E).^[1]^ While the acute phase of the illness usually lasts for several weeks, a significant proportion of cases remain of unknown origin. They become diagnostic and therapeutic challenge for clinicians. These cases represent a diagnostic and therapeutic challenge, especially when conventional serological and biochemical markers are negative.

Recently, there has been an increase in cases of acute hepatitis of unknown cause, referred to in medical literature as acute hepatitis of unknown origin (AHUO). According to data from the World Health Organization, as of 2022, there were 572 reported cases of AHUO worldwide.^[2]^ Out of all the reported cases, only 26 were in the Balkan region. Despite the rising frequency of AHUO, a clear therapeutic approach or standardized treatment protocol has yet to be established in medical literature. We present a case of AHUO in a 38-year-old male from the Balkan region, distinguished by a markedly severe transaminase elevation (alanine aminotransferase [ALAT] > 4500 U/L). Despite this severe presentation, the patient experienced rapid and complete resolution with only conservative, supportive therapy. This report details the extensive negative diagnostic workup and discusses the clinical reasoning for deferring an invasive liver biopsy in the context of spontaneous, rapid improvement.

## 2. Clinical case

We present a clinical case of a 38-year-old male who was admitted to Clinic of Infectious Diseases on July 10, 2025. He presented with general fatigue, a fever of up to 37.7°C, abdominal discomfort, and a loss of appetite.

On July 7, 2025, he noticed yellowing of his sclera, darkening of his urine, and pale stools.

Upon admission, his laboratory tests showed significantly elevated liver enzymes:

- Total bilirubin: 156 µmol/L.
- Direct bilirubin: 116 µmol/L (normal range up to 21).
- Aspartate aminotransferase: 1001 U/L (normal range up to 40).
- Alanine aminotransferase: 4559 U/L (normal range up to 41).
- Gamma-glutamyl transferase: 135 U/L (normal range up to 60).
- Alkaline phosphatase: 94 U/L (normal range up to 129).

**Figure 1.**
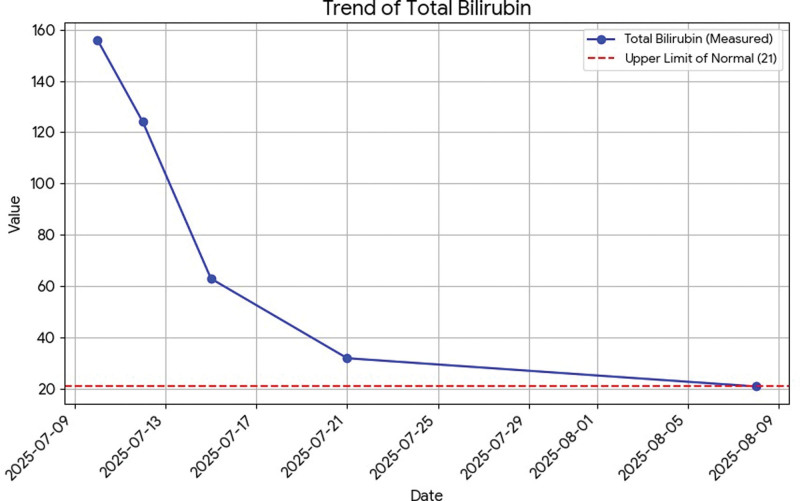
Dynamics of total bilirubin.

**Figure 2.**
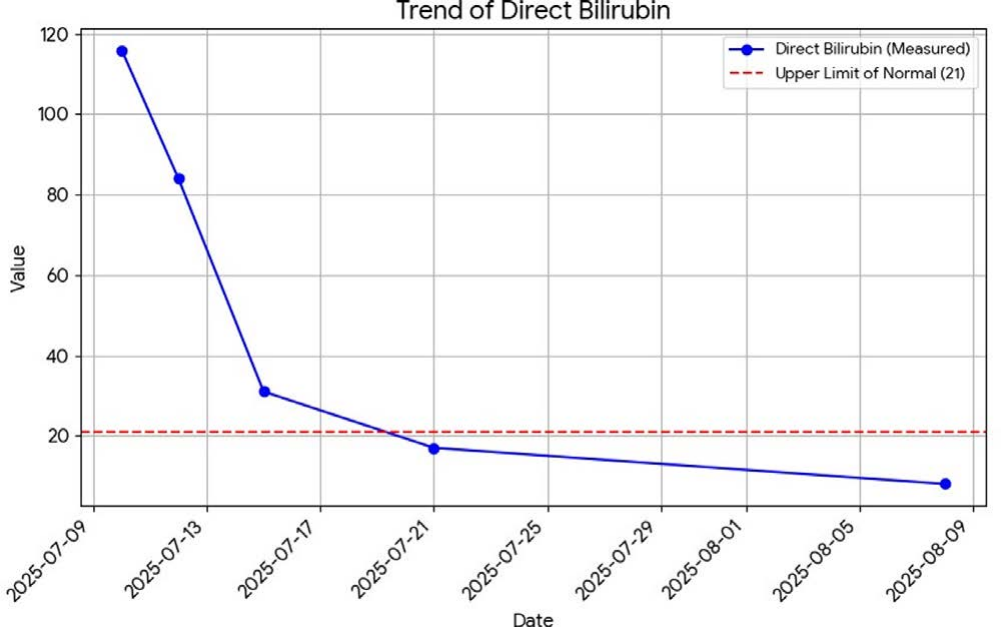
Dynamics of direct bilirubin.

**Figure 3.**
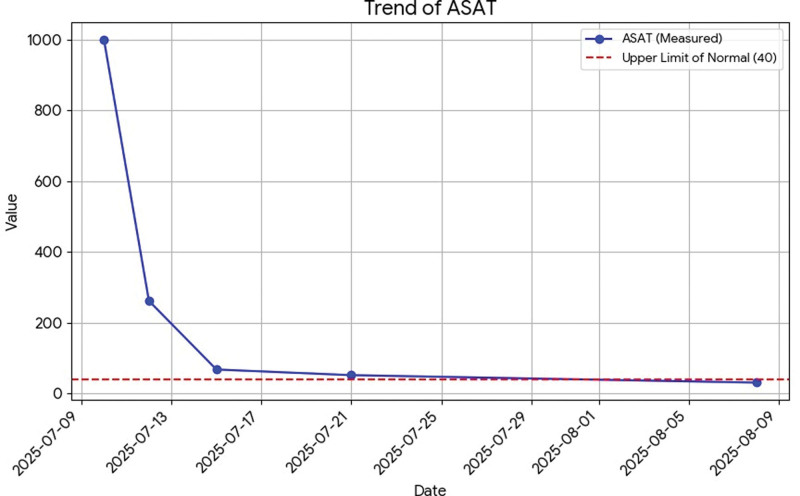
Dynamics of ASAT. ASAT = aspartate aminotransferase.

**Figure 4.**
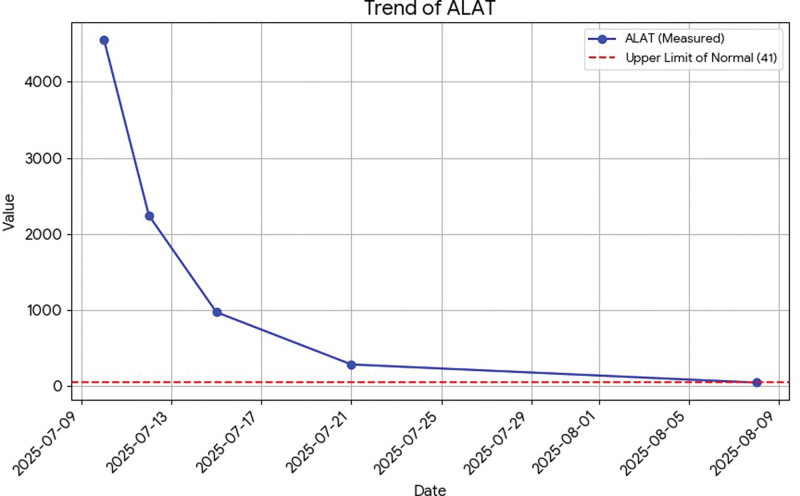
Dynamics of ALAT. ALAT = alanine aminotransferase.

**Figure 5.**
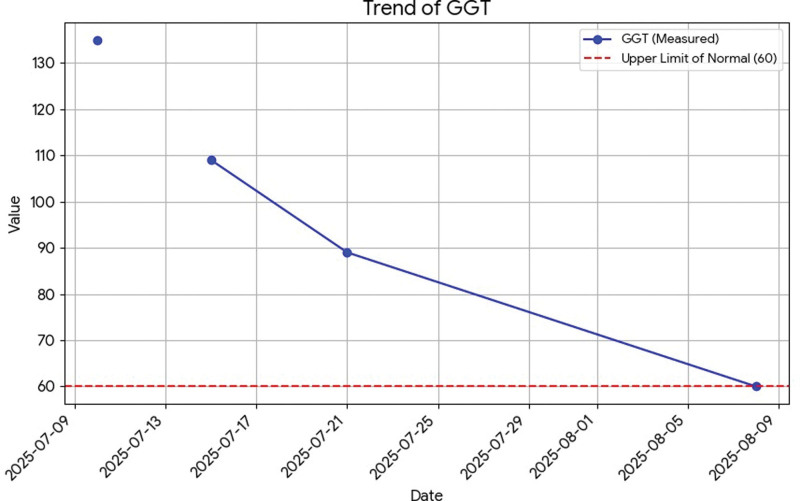
Dynamics of GGT. GGT = gamma-glutamyl transferase.

**Figure 6.**
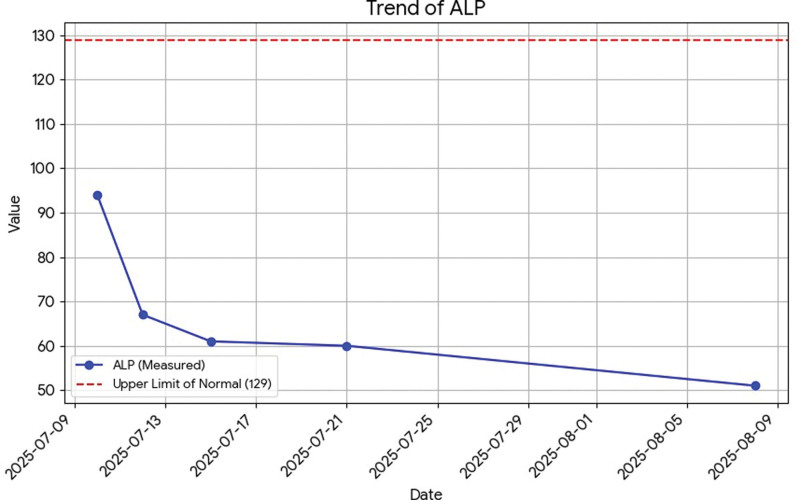
Dynamics of alkaline phosphatase.

All liver tests are presented in dynamic on Figures 1–6.

The patient denied contact with sick people, any blood transfusions or manipulations in the last 6 months, and recent travel abroad. His alcohol consumption is infrequent, last intake of approximately 300 mL ouzo several day prior his laboratory results.

His only comorbidity was arterial hypertension on stable therapy. He has no history of harmful habits or allergies.

An abdominal ultrasound revealed no signs of mechanical jaundice, the liver appeared a little bit enlated.

Further investigations were conducted to rule out other causes. All test are showed on Table 1.

**Table 1:** Results of screening for intoxication and infectious agents.

Test category	Results
Creatine phosphokinase (CPK)	Within reference limits.
Toxicological analysis (blood)	Negative: methanol, amphetamines, methamphetamine, ecstasy (3,4-methylenedioxymethamphetamine), opiates (heroin, morphine, codeine), tetrahydrocannabinol, methadone, cocaine, tricyclic antidepressants, benzodiazepines, and barbiturates.
	Positive: bisoprolol and its metabolite, ibuprofen, and caffeine and its metabolite.
	Conclusion: no evidence of exogenous intoxication requiring specific detoxification therapy.
Hepatitis markers	Negative for anti-HAV IgM, anti-HAV IgG, HBsAg, HBeAg, anti-HBeAg, anti-HBc IgM, anti-HBc total, anti-HCV, and anti-HEV IgM.
Other infectious disease tests	Negative for *Coxiella burnetti* (Q fever), Ebstein–Barr virus IgM, cytomegalovirus IgM, adenovirus IgM, Hanta virus IgM, herpes simplex, and leptospirosis.
Throat swab culture	Did not isolate any pathogenic aerobic bacteria or *Candida* spp.

Anti-HAV = anti-hepatitis A virus, anti-HCV = anti-hepatitis C virus, anti-HEV = anti-hepatitis E virus.

The patient was started on intravenous treatment, which included glucose solutions, vitamins (C and Ca gluconate), pantoprazole, ademetionine, and L-ornithine L-aspartate. This therapy resulted in a good response. He was discharged and referred to the Clinic of Gastroenterology for further evaluation to rule out other potential causes for the elevated transaminase activity.

He was admitted to the Clinic of Gastroenterology at University Hospital “St. Ivan Rilski” on July 15, 2025. Key lab results are showed on Table 2.

**Table 2:** Results of metabolic and immunological workup.

Test category	Test name	Result (reference range/normal)
Metabolic/iron panel	Ceruloplasmin	0.14 g/L (0.15–0.45 g/L)
	Transferrin saturation (% Tf)	40.4% (male: 30–40%)
	Ferritin	592 (male: 40–280)
	Serum iron	25.5 µmol/L (11–30 µmol/L)
Wilson disease workup	Ophthalmological exam	No evidence of a Kayser–Fleischer ring
	24-h Cupruria	0.3 mmol/24 h (negative)
Immunological tests	Immunoglobulins	IgA: slightly elevated. IgG & IgM: within normal limits.
	AHA	1:80
	Autoantibody panel	All Negative: ASMA, AMA, AMA-M2, anti-LKM, 3E (BPO), Sp100, PML, gp210, LC1, SLA/LP, SS-A, Ro52, Scl-70, CENP A, CENP B, PGDH

Given the patient’s rapid and significant improvement, and after a discussion of the procedure’s risks (such as bleeding) versus its limited benefits in his resolving condition, the patient expressed a clear preference to postpone the invasive test. The joint clinical team will instead monitor his liver enzymes every 2 weeks for 3 months, reserving a biopsy only if a potential relapse occurs.

The patient was discharged with a significant decrease in transaminase activity due to the intravenous therapy. Subsequent follow-up showed a gradual normalization of his liver enzymes within approximately 1 month of the initial lab tests and 1 more time negative results for hepatitis viruses.

## 3. Discussion

AHUO is characterized by acute necrosis and inflammation of hepatocytes.^[3]^ Although hepatotropic viruses account for about 95% of cases, other viral agents such as herpes simplex, Epstein–Barr, and cytomegalovirus may also cause acute hepatitis.^[4]^ In our patient, all of these were tested and excluded. Possible drug- or toxin-induced causes were also investigated, but toxicological screening was negative. Following the recommendations by Kwong, we performed a comprehensive evaluation for autoimmune liver disease and rarer metabolic disorders, including Wilson disease and hemochromatosis.^[5]^ All were ruled out in our clinical case.

The literature describes similar clinical cases. One report presented an adult female with AHUO in whom corticosteroid therapy was considered.^[6]^ They withheld due to because of the histological result that suggested a possible infectious origin. Another case described by Palla et al involved acute hepatitis in a 40-year-old woman shortly after receiving the Pfizer vaccine.^[7]^ Infectious and autoimmune causes were excluded, but a nonspecific positive ANA was noted. This case report adds to the limited literature on adult AHUO by highlighting 2 key features: the extreme severity of the initial transaminase elevation (ALAT > 4500 U/L) and the rapid, complete resolution with only conservative supportive therapy.

As in the AHUO reports, our diagnostic process involved an exhaustive and ultimately negative workup, ruling out common viral, toxicological, autoimmune, and metabolic causes, including Wilson disease. However, a key distinguishing feature of our case was the degree of hepatocellular injury. Other published adult cases, such as those considering corticosteroid therapy or noting nonspecific histological findings, reported significantly lower enzyme elevations.

In our case, we adopted a conservative therapeutic strategy with supportive measures, including daily glucose infusions combined with hepatoprotective agents such as silymarin, vitamin C, and ademetionine.^[8]^ The patient responded well, with normalization of liver enzymes and no need for further intervention. This favorable outcome supports the idea that supportive therapy may be sufficient in adults with AHUO, even when liver enzyme elevations are markedly high.

It is worth noting that the disease course can differ in children. The literature review by Namakin et al discusses the rising number of AHUO cases in children, with 1010 cases described by 2022.^[9]^ Five percent of children required a liver transplant, and 2% resulted in death. This shows that an unspecified hepatotropic virus can cause a more severe reaction in children than in adults.

In our case and the 2 other reviewed, supportive therapy was effective. It leads to a full recovery without the need for more aggressive interventions. This suggests that while AHUO can be severe, a conservative and watchful approach can lead to a positive outcome in adult patients.

## 4. Conclusion

We present a case of AHUO in an adult male in whom we ruled out numerous possible causes of acute liver injury. The most common causes of acute liver injury are viral hepatitis and drug-induced hepatitis, and we performed extensive laboratory and toxicological analyses on our patient.

Despite markedly elevated transaminases, he responded well to supportive therapy commonly used in acute viral hepatitis, with complete normalization of liver enzymes during 1-month follow-up.

## Author contributions

**Conceptualization:** Petar Trifonov, Cvetelina Marinova, Anelia Zasheva, Dimitar Komitov, Rosen Nikolov, Raynichka Mihaylova- Garnizova.

**Data curation:** Anelia Zasheva, Dimitar Komitov.

**Supervision:** Cvetelina Marinova, Rosen Nikolov, Raynichka Mihaylova- Garnizova.

**Validation:** Cvetelina Marinova, Dimitar Komitov, Rosen Nikolov, Raynichka Mihaylova- Garnizova.

**Visualization:** Cvetelina Marinova, Dimitar Komitov.

**Writing – original draft:** Petar Trifonov, Donika Todovichin.

**Writing – review & editing:** Petar Trifonov, Donika Todovichin, Cvetelina Marinova, Anelia Zasheva, Dimitar Komitov.
